# Maternal HIF1A-IGFBP2 dysregulation links endometriosis to gestational diabetes: a prospective cohort study

**DOI:** 10.3389/fendo.2026.1948558

**Published:** 2026-09-24

**Authors:** Jiao Fan, Xiaoying He

**Affiliations:** Department of Family Planning, Shanghai First Maternity and Infant Health Hospital, Shanghai, China

**Keywords:** biomarker, central carbon metabolism, endometriosis, gestational diabetes mellitus, HIF1A, IGFBP2, single-cell RNA sequencing

## Abstract

**Background:**

Endometriosis (EM) may be associated with adverse pregnancy outcomes, but its relationship with gestational diabetes mellitus (GDM) and circulating metabolic biomarkers remains incompletely defined. This study evaluated temporal associations and exploratory discrimination of circulating HIF1A and IGFBP2 with maternal glycemia and criteria-defined GDM.

**Methods:**

This prospective single-center cohort included 200 pregnant women with laparoscopically diagnosed EM and 200 controls without known or clinically detected EM. Serum HIF1A and IGFBP2 were measured at enrollment (6–7 weeks of gestation) and again during mid-pregnancy (24–28 weeks). Early measurements preceded the later GDM assessment; the mid-pregnancy blood sample and the 75-g OGTT were both obtained within the 24–28-week window. GDM was defined using IADPSG fasting-glucose and 75-g OGTT thresholds applied directly to the raw glucose data. Spearman rank correlation, age- and BMI-adjusted logistic regression, exploratory ROC analysis with 2,000 stratified bootstrap resamples, and bootstrap optimism correction were performed.

**Results:**

Criteria-defined GDM occurred in 69/200 women with EM (34.5%) and 27/200 controls (13.5%; RR 2.56, 95% CI 1.71–3.81; P<0.001). HIF1A and IGFBP2 concentrations were lower in the EM group at both pregnancy time points. Within the EM cohort, Spearman rank correlations with mid-pregnancy fasting blood glucose were −0.68 for early-pregnancy HIF1A, −0.50 for early-pregnancy IGFBP2, −0.54 for mid-pregnancy HIF1A, and −0.89 for mid-pregnancy IGFBP2 (all P<0.001). In adjusted logistic models, lower early HIF1A, early IGFBP2, mid-pregnancy HIF1A, and mid-pregnancy IGFBP2 were associated with GDM, with ORs of 0.922, 0.920, 0.798, and 0.941, respectively. Individual-marker AUCs were 0.844 and 0.688 in early pregnancy and 0.871 and 0.889 in mid-pregnancy. Combined-model AUCs were 0.856 and 0.888, with optimism-corrected AUCs of 0.845 and 0.882.

**Conclusions:**

In this cohort, EM was associated with a higher frequency of criteria-defined GDM and lower circulating HIF1A and IGFBP2 concentrations. These biomarkers showed promising exploratory internal diagnostic performance, but the findings are observational and require external validation.

## Introduction

Endometriosis was a common chronic disease among women of childbearing age. Its pathological characteristic was the presence of endometrial-like tissue outside the uterine cavity, commonly found in pelvic structures such as the ovaries, peritoneum, Douglas pouch, and uterine sacral ligaments. This disease was highly associated with infertility, affecting approximately 50% of affected patients (1). The pelvis was the most common site for endometriosis lesions, and its structure was continuously exposed to peritoneal fluid rich in cytokines secreted by the lesions. Studies have shown that peritoneal fluid from endometriosis patients could significantly promote the proliferation of endometrial stromal cells *in vitro* (2). A detailed analysis of peritoneal fluid components revealed that, compared to patients without endometriosis, patients with endometriosis had significantly elevated levels of insulin-like growth factor-I (IGF-I) in their peritoneal fluid, while levels of insulin-like growth factor-binding protein-3 (IGFBP-3) and the relative ratio of IGFBP2 (the ratio of IGFBP2 intensity to total IGFBP intensity) were significantly reduced (3).

It is worth noting that serum IGFBP2 levels have been widely proven to be closely related to metabolic health, and its reduced concentration is an important risk factor for obesity, metabolic syndrome, insulin resistance, type 2 diabetes mellitus (T2DM), and gestational diabetes mellitus (GDM) (4, 5).

Beyond its classical role in hypoxia, Hypoxia-inducible factor 1-alpha (HIF1A) serves as a critical upstream governor of central carbon metabolism, dictating cellular substrate selection and glycolytic programmatic switching (6, 7). Accumulating clinical and mechanistical evidence has established that the functional impairment or maladaptation of HIF1A is profoundly intertwined with metabolic syndromes, directly driving the onset of local glucose utilization bottlenecks and distal insulin resistance (8, 9). Intriguingly, either localized transcriptional failure or atypical ROS-mediated hyper-stabilization of the HIF1A machinery can severely disrupt glucose uptake and downstream carbon processing, as manifested in congenital metabolic disorders or chronic microenvironmental stressors (10). Therefore, decoding the cell-type-specific dysregulation of the HIF1A-driven energetic network provides pivotal molecular insights into the systemic homeostatic subversion and glucose intolerance under pathological conditions.

HIF1A and the IGF signaling system have long been recognized to form a reciprocal positive feedback loop, where HIF1A directly transactivates IGF ligands and their binding proteins (IGFBPs), while IGF activation conversely stabilizes HIF1A translation via downstream cascade (11). However, whether this classic synergetic circuit is disrupted within the cellular compartments of endometriosis remains unexplored.

Given this background, a critical knowledge gap remains: whether the HIF1A–IGFBP2 axis is associated with maternal metabolic adaptation during pregnancy in women with endometriosis. In this study, we used public single-cell RNA-sequencing data (GSE213216) as exploratory biological context and examined a prospective clinical cohort of 400 pregnant women. Clinical analyses evaluated whether early-pregnancy (6–7 weeks) and mid-pregnancy (24–28 weeks) circulating HIF1A and IGFBP2 were associated with maternal glycemic measures and criteria-defined GDM. Early biomarker measurements preceded the later diagnostic window, whereas mid-pregnancy measurements were obtained within the same gestational window as the 75-g OGTT; therefore, the latter are contemporaneous diagnostic associations rather than prospective prediction. The study was designed to generate clinically testable hypotheses, not to establish a causal lesion-to-maternal metabolic mechanism or a validated biomarker. Our findings offer a novel, mechanistically anchored strategy for the early interception of GDM in patients with a history of endometriosis.

## Methods

### Public scRNA-seq data acquisition and preprocessing

Public single-cell RNA sequencing (scRNA-seq) datasets of human ectopic endometriotic lesions (EM) and normal control endometrium (NE) were retrieved from the Gene Expression Omnibus (GEO) database under accession number GSE213216. Metadata verification confirmed that all enrolled donor patients were hormone-therapy-naïve (free of exogenous oral contraceptives, progestins, or GnRH agonists) for at least 3 to 6 months prior to surgical tissue collection, ensuring an unconfounded endogenous state. A total of 6 individual tissue donors—3 ectopic lesion samples (EM1, EM2, EM3) and 3 normal control tissue samples (NE1, NE2, NE3)—were incorporated into downstream bioinformatic processing. Bioinformatic analyses were executed using the Seurat R package (version 4.3.0) in R environment (version 4.2.2). To remove low-quality cells, damaged cells, or potential doublets, strict quality control (QC) filtering was applied. Briefly, individual cells were retained only if they met the following criteria: (1) number of detected genes (nFeature_RNA) between 500 and 6,000; (2) total unique molecular identifier (UMI) counts (nCount_RNA) between 1,000 and 40,000; and (3) percentage of mitochondrial gene expression (percent.mt) < 15%. Following global log-normalization (NormalizeData with a scale factor of 10,000), the top 2,000 highly variable genes (HVGs) were identified using the FindVariableFeatures function with the ‘vst’ selection method.

### Integration, dimensional reduction, and confounding control

To eliminate potential batch effects and inter-patient donor heterogeneity, as well as variations stemming from tissue sampling sites or subtle menstrual cycle phase differences, data integration across all 6 donors was executed using the Harmony algorithm (version 0.1.1). Linear dimensional reduction via Principal Component Analysis (PCA) was performed on the integrated HVG matrix. The top 30 principal components (PCs) were selected based on the Elbow plot inflection point and utilized for non-linear dimensional projection via Uniform Manifold Approximation and Projection (UMAP).Unsupervised graph-based clustering was implemented using the FindNeighbors (top 30 PCs) and FindClusters functions (resolution parameter set to 0.5). Major functional cell lineages were annotated based on the expression of canonical biological markers: EPCAM and KRT18 for Epithelial cells; DCN and PDGFRB for Stromal cells; PECAM1 and VWF for Endothelial cells; CD68 and CD163 for Macrophages; and CD3D and PTPRC for Immune cells. Exact cell counts per annotated lineage and individual donor were quantified and summarized (**Supplementary Table S1**).

### Co-expression profiling and gene set enrichment analysis

To evaluate the cellular co-regulation between HIF1A and IGFBP2, Spearman’s rank correlation analysis was computed across individual cells strictly within the functional parenchymal lineages (epithelial and stromal compartments) of the EM ectopic group. To resolve downstream metabolic functional remodeling, cells within the EM parenchymal pool were stratified into IGFBP2_High and IGFBP2_Low subsets using the median expression value of IGFBP2 as the cutoff threshold. Differentially expressed genes (DEGs) between these two subsets were generated using the FindMarkers function with the Wilcoxon rank-sum test. Gene Set Enrichment Analysis (GSEA) was performed against the Kyoto Encyclopedia of Genes and Genomes (KEGG) metabolic reference pathways using the clusterProfiler R package (version 4.6.0). Pathways with an adjusted P-value < 0.05 and a false discovery rate (FDR) < 0.25 were deemed statistically significant. Expression distributions of key central carbon metabolism and rate-limiting enzyme genes (PKM, LDHA, SDHA, MDH2) were visualized using target-specific single-cell violin plots (VlnPlot).

### Patients

This study targeted women with singleton pregnancies who registered at the obstetrics outpatient clinic during the early stages of pregnancy (6–7 weeks of gestation). All pregnant women were confirmed to have a single live fetus *in utero* via ultrasound examination. Inclusion criteria included: age 20–40 years, pre-pregnancy BMI 18.5–23.9 kg/m². Participants were divided into two groups based on the diagnosis of endometriosis (EMs): EMs group (n=200): Pre-pregnancy laparoscopic surgery confirmed endometriosis (rASRM stage I–IV); Control group (n=200): No history of endometriosis, and EMs was ruled out through detailed medical history records, pre-pregnancy or early pregnancy gynecological examinations, and pelvic ultrasound. Exclusion criteria: History of pre-pregnancy diabetes, gestational diabetes mellitus (GDM), polycystic ovary syndrome (PCOS), or family history of diabetes; Multiple pregnancies; Severe liver or kidney dysfunction, autoimmune diseases, active malignant tumors, or concurrent adenomyosis; Current use of medications affecting glucose and lipid metabolism (e.g., corticosteroids, statins, thiazolidinediones, or metformin); Previous history of GDM (to avoid interference from prior medical history);Smokers or heavy drinkers. This study was approved by the Ethics Committee of the Obstetrics and Gynecology Hospital Affiliated with Tongji University (Approval No. KS24466) and registered with the Chinese Clinical Trial Registry (Registration No. ChiCTR2500095800) on January 13, 2025. This study was conducted from February 2025 to February 2026. All clinical procedures and sample collection were conducted strictly in accordance with the guidelines and ethical standards of the Declaration of Helsinki. All enrolled participants provided written informed consent prior to their inclusion in the study.

### Blood sample testing

During early pregnancy (6–7 weeks), fasting venous blood was collected at enrollment for serum HIF1A, IGFBP2, fasting glucose, HbA1c, lipid variables, and FFA. During mid-pregnancy (24–28 weeks), a second fasting venous sample was collected for the same circulating biomarkers and metabolic assays. The routine 75-g OGTT was performed within the same 24–28-week window. The supplied records do not report the exact number of days between the mid-pregnancy blood draw and the OGTT, or their within-visit order. Accordingly, early-pregnancy biomarker measurements preceded the later GDM assessment, whereas mid-pregnancy biomarker measurements should be interpreted as contemporaneous with diagnostic testing rather than as prospective predictors. GDM was defined using IADPSG criteria: fasting blood glucose ≥ 5.1 mmol/L or 1-hour blood glucose ≥ 10.0 mmol/L or 2-hour blood glucose ≥ 8.5 mmol/L; meeting any one threshold was sufficient for a GDM diagnosis.

### ELISA

After collection, the patient’s blood was stored in a -80 °C freezer. Serum concentrations of Hypoxia-inducible factor 1-alpha (HIF1A) were measured using a commercially available Human HIF1A ELISA Kit (Catalog No. ab229433, abcam, Cambridge, UK). Serum concentrations of insulin-like growth factor binding protein 2 (IGFBP2) were measured using a commercially available Human IGFBP2 ELISA Kit (Catalog No. ab272207, abcam). All experimental procedure were conducted strictly according to the kit instructions.

### Biochemical molecular detection

All biochemical parameters were measured in fasting venous blood samples using standard clinical automated assays in the clinical laboratory of Obstetrics and Gynecology Hospital affiliated with Tongji University. Total cholesterol (TC) and high-density lipoprotein cholesterol (HDL-C) were measured by enzymatic colorimetric assays. For TC, cholesterol esters were hydrolyzed by cholesterol esterase to free cholesterol, which was then oxidized by cholesterol oxidase to produce hydrogen peroxide. The hydrogen peroxide was quantified via a peroxidase-catalyzed reaction with a chromogen. HDL-C was measured by a direct homogeneous method. Analyses were performed on an automated analyzer (Roche Cobas c702, USA). Free fatty acids (FFA) were measured by an enzymatic method using a commercial kit. Serum FFAs were converted to acyl-CoA derivatives, which are oxidized to generate hydrogen peroxide, measured by a Trinder reaction. Fasting plasma glucose (FPG) was measured by the glucose oxidase method. Glucose was oxidized by glucose oxidase to produce gluconic acid and hydrogen peroxide, which is then measured spectrophotometrically. Glycated hemoglobin (HbA1c) was quantified by high-performance liquid chromatography (HPLC) on a analyzer (Bio-Rad D-100, USA). HbA1c is reported as a percentage of total hemoglobin.

### Statistical analysis

Comparison of baseline characteristics: Quantitative data between the two groups were analyzed using t-tests or Mann-Whitney U tests, while categorical data were analyzed using chi-square tests or Fisher’s exact tests. The incidence rate of GDM (%), relative risk (RR), and 95% confidence intervals (CI) between the two groups were calculated. Spearman’s rank correlation analysis was used to examine the relationship between blood HIF1A and IGFBP2 levels and fatty acid levels and fasting blood glucose levels. Binary logistic regression was used to validate the association between HIF1A and IGFBP2 and the occurrence of GDM. ROC curves were plotted to assess the predictive efficacy of serum HIF1A or IGFBP2 for the subsequent occurrence of GDM.

## Results

### Single-cell transcriptomic atlas map identifies cell-type-specific silencing of HIF1A and IGFBP2 within ectopic lesions

To capture the cellular heterogeneity and cell-type-specific molecular disturbances characterizing endometriosis, we carried out high-resolution single-cell RNA sequencing (scRNA-seq) on tissues derived from ectopic lesions (EM group) and normal endometrium (NE group). Unsupervised UMAP embedding clustered the comprehensive cellular pool into five highly annotated specific lineages based on established canonical markers: Endothelial cells (orange), Epithelial cells (red), Immune cells (blue), Macrophages (purple), and Stromal cells (green), alongside a minor fraction of Unidentified functional entities (grey) (**Figure 1A**). We next projected the spatial transcriptomic topography of IGFBP2 and HIF1A across these distinct cellular niches using single-cell Feature Plots (**Figure 1B**). Intriguingly, comparative dot-plot profiling across group-stratified cell types revealed a selective, distinct transcriptional deficit of both HIF1A and IGFBP2. This depletion was prominently restricted within the functional lineages—primarily epithelial and stromal cells—of the EM lesions compared to their NE counterparts (**Figure 1B**). To verify this local down-regulation with statistical rigor, we performed single-cell quantitative violin plot analyses, which established a profound, synchronized compression of endogenous IGFBP2 and HIF1A expression levels inside the ectopic microenvironment compared to healthy tissues (P < 0.001; **Figure 1C**).

**Figure 1:**
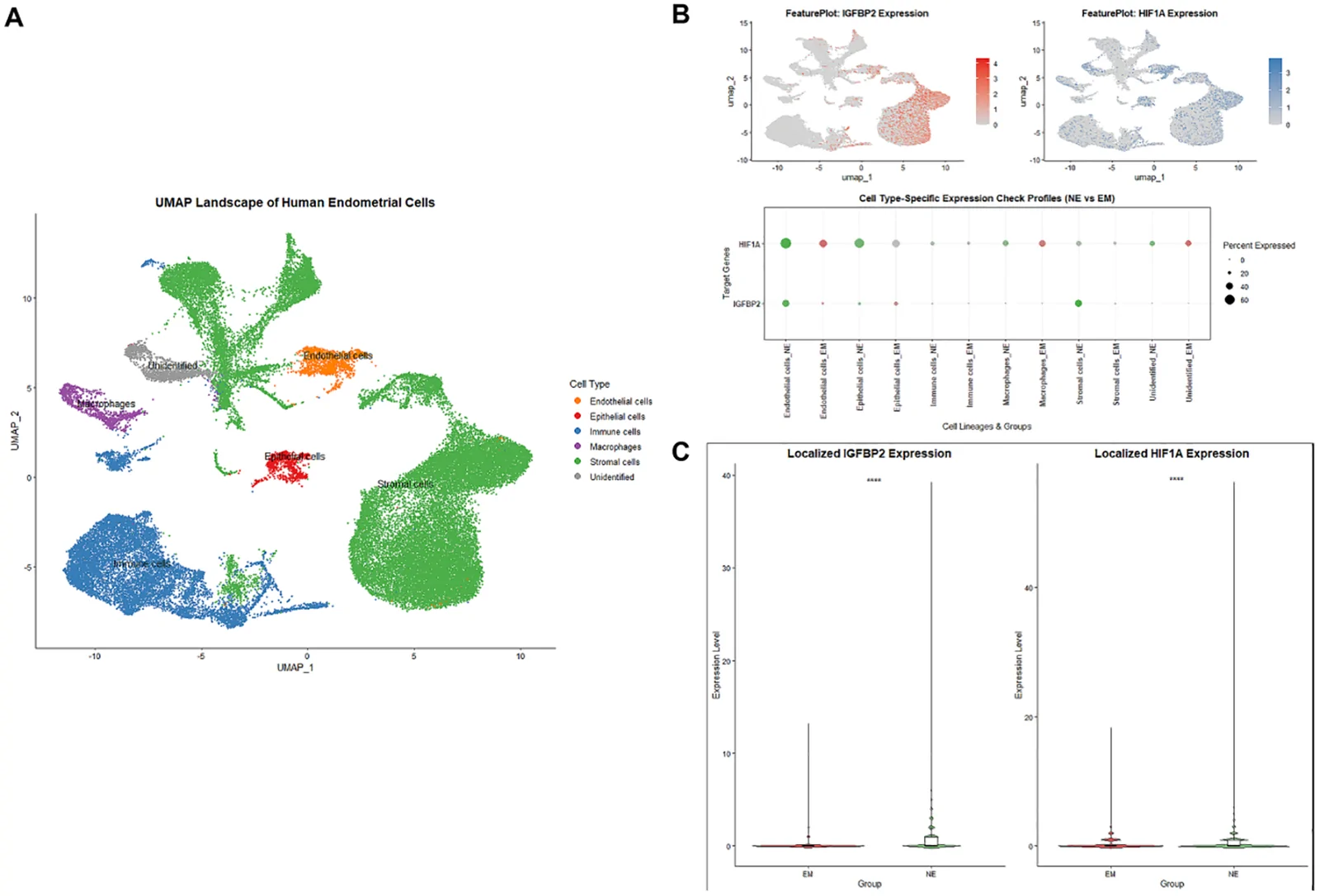
High-resolution single-cell transcriptomic atlas and cell-type-specific HIF1A/IGFBP2 expression in human endometrial and ectopic lesion samples. **(A)** UMAP Landscape of Human Endometrial Cells: Two-dimensional UMAP visualization plot showing the unsupervised clustering and distinct spatial segregation of primary cell types isolated from human endometrium (NE) and ectopic lesion (EM) tissues. Major annotated lineages are color-coded: Endothelial cells (orange), Epithelial cells (red), Immune cells (blue), Macrophages (purple), Stromal cells (green), and Unidentified functional clusters (grey). **(B)** Cell-Type Topography and Expression Profiles: (Top) Feature Plots illustrating the transcriptomic density and localized spatial distribution of IGFBP2 (left) and HIF1A (right) across the UMAP landscape. (Bottom) Comparative dot-plot matrix mapping cell-type-specific expression metrics between NE and EM cohorts. Dot size indicates the percentage of cells expressing the target gene, and color intensity corresponds to the normalized expression scale, highlighting a localized depletion in EM functional lineages. **(C)** Quantitative Single-Cell Expression Verification: Quantitative violin plots demonstrating a severe transcriptomic reduction of endogenous IGFBP2 (left) and HIF1A (right) levels inside the local EM microenvironment compared with healthy NE control tissues.

### The coupled HIF1A-IGFBP2 axis controls a downstream metabolic cascade and rate-limiting central carbon machinery

Given the synchronous tissue-level downregulation of HIF1A and IGFBP2, we hypothesized that they operate as an interconnected functional circuit rather than independent nodes. Linear regression analysis applied to individual functional cells within the EM microenvironment unveiled a remarkably robust and statistically clean positive co-expression correlation between HIF1A and IGFBP2 transcript abundance (r = 0.42, P = 9e-08; **Figure 2A**), strongly validating the existence of a coupled HIF1A-IGFBP2 coregulatory engine under pathological stress. To resolve the biological sequelae driven by the disruption of this interlinked axis, we stratified the EM cellular pool into IGFBP2_High and IGFBP2_Low sub-populations. Gene Set Enrichment Analysis (GSEA) applied to these groups revealed a devastating, multi-pathway paralysis of central energy-harvesting programs. The top enriched metabolic cascades—namely “Carbon metabolism” (NES = 1.29, p.adj = 0.010), “Glycolysis/Gluconeogenesis” (NES = 1.3, p.adj = 0.043), and the “Citrate cycle (TCA cycle)” (NES = 1.31, p.adj = 0.104)—displayed prominent, synchronized down-regulation in the IGFBP2_Low cellular compartment (**Figure 2B**). To trace the downstream executioners of this metabolic block, we quantified the transcription of individual rate-limiting metabolic enzymes across the subsets (**Figure 2C**). In the cytoplasmic glucose-processing sector, drivers like Pyruvate Kinase M1/2 (PKM) and Lactate Dehydrogenase A (LDHA) were dramatically squelched toward a baseline zero state in the IGFBP2_Low cohort. Concurrently, within the mitochondrial oxidative phosphorylation apparatus, the transcription of core tricarboxylic acid cycle hubs, including Succinate Dehydrogenase Complex Subunit A (SDHA) and Malate Dehydrogenase 2 (MDH2), was almost entirely flattened in the low-expression cluster.

**Figure 2:**
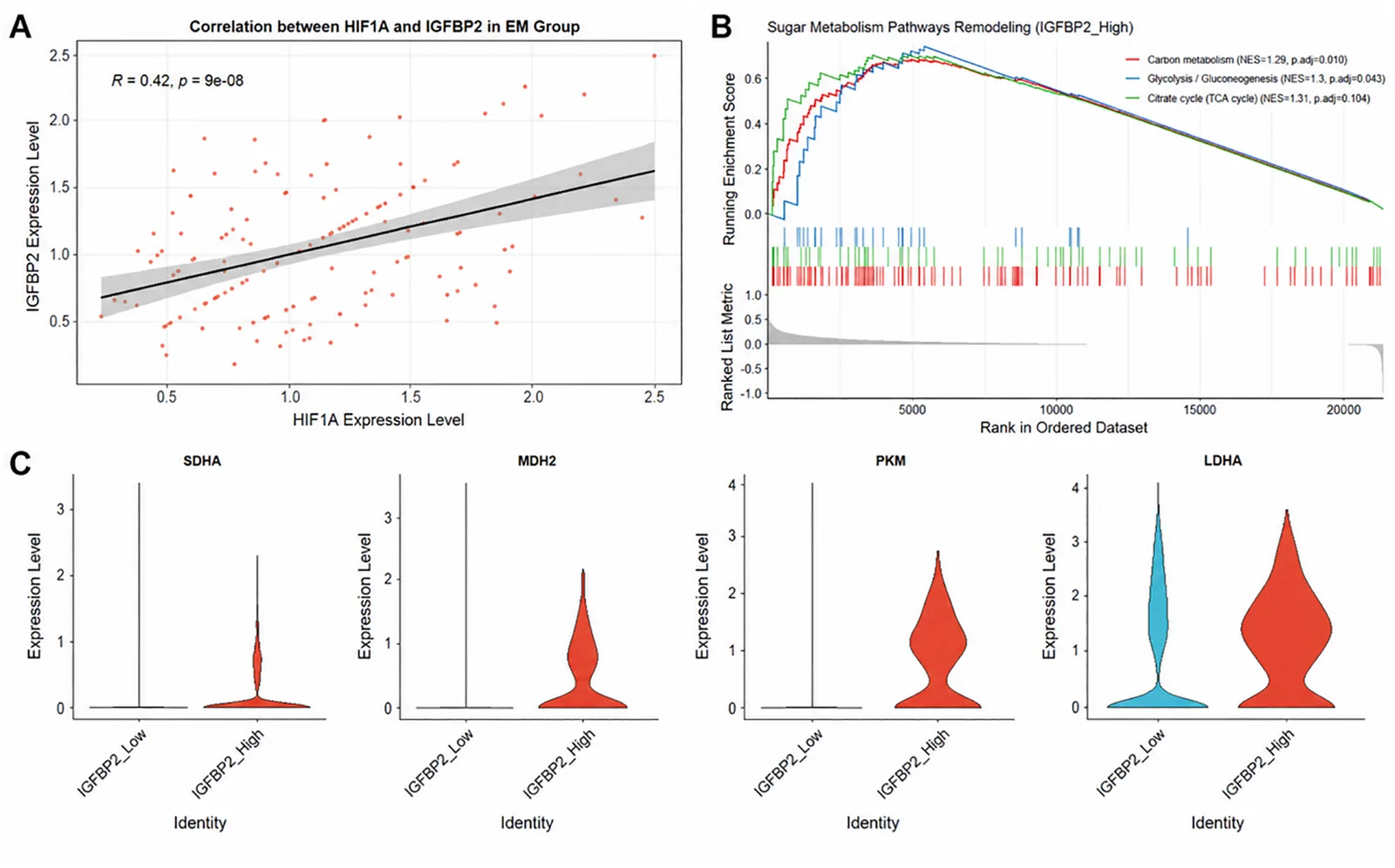
Localized co-expression coupling of the HIF1A-IGFBP2 axis and subsequent downregulation of central carbon processing networks in ectopic lesions. **(A)** Linear Co-expression Regression within Ectopic Cells: Two-variable single-cell scatter plot verifying a robust, statistically highly significant positive correlation between HIF1A and IGFBP2 transcript abundance specifically within individual functional cells of the EM lesion niche (r = 0.42, P = 9e-08), supporting their shared pathological coregulation. **(B)** Pathway Enrichment via GSEA Mapping: (Left) Glucose Metabolism Dotplot showing the multi-pathway down-regulation of primary metabolic pathways including the Citrate cycle (TCA cycle), Carbon metabolism, and Glycolysis/Gluconeogenesis cascades. Circle size reflects gene counts, and the color bar indexes the statistical P-value. (Right) GSEA running enrichment score curves demonstrating the synchronized negative enrichment profiles of sugar processing programs in the IGFBP2_Low cellular cohort. **(C)** Transcriptional Ablation of Rate-Limiting Metabolic Enzymes: Target-specific single-cell violin plots tracking individual core enzymes governing mitochondrial oxidative respiration (SDHA, MDH2) and cytoplasmic glycolytic processing (PKM, LDHA) stratified between IGFBP2_Low (blue) and IGFBP2_High (red) subsets.

### Comparison of molecular markers between the two groups

There were no statistically significant differences between EM and control groups in age, BMI, gravidity, or parity (**Table 1**).

**Table 1:** Baseline characteristics of the two groups.

Variables	EM group (n=200)	Control group (n=200)	P-value
Age (years)	29.50 ± 5.00	29.54 ± 5.13	0.94
BMI (kg/m²)	20.79 ± 0.92	20.65 ± 0.92	0.15
Gravidity (n)	2.49 ± 1.31	2.35 ± 1.21	0.27
Parity (n)	0.64 ± 0.63	0.67 ± 0.67	0.70
rASRM stage I–II	156 (78%)	/	
rASRM stage III–IV	44 (22%)	/	

Data are shown as mean ± SD or n (%). Group comparisons used two-sample t-tests unless otherwise indicated.

ASRM, American Society for Reproductive Medicine.

Early-pregnancy fasting glucose, HbA1c, lipid variables, and FFA were similar between groups (**Table 2**). Early-pregnancy HIF1A and IGFBP2 concentrations were lower in the EM group than in controls (both P<0.001).

**Table 2:** Metabolic variables and circulating biomarkers in early pregnancy.

Variables	EM group (n=200)	Control group (n=200)	P-value
FBG (mmol/L)	5.10 ± 0.45	5.02 ± 0.43	0.10
HbA1c (%)	4.91 ± 0.45	4.84 ± 0.41	0.10
TC (mmol/L)	3.10 ± 0.35	3.10 ± 0.38	0.93
TG (mmol/L)	0.68 ± 0.20	0.70 ± 0.23	0.42
HDL (mmol/L)	1.11 ± 0.16	1.12 ± 0.18	0.34
LDL (mmol/L)	1.78 ± 0.26	1.77 ± 0.24	0.68
FFA (mmol/L)	0.35 ± 0.12	0.37 ± 0.21	0.14
HIF1A (pg/mL)	141.91 ± 23.53	347.86 ± 41.86	<0.001
IGFBP2 (pg/mL)	13.48 ± 7.73	32.32 ± 5.04	<0.001

FBG, fasting blood glucose; HbA1c, glycated hemoglobin; TC, total cholesterol; TG, triglyceride; HDL, high-density lipoprotein; LDL, low-density lipoprotein; FFA, free fatty acid.

At mid-pregnancy, the EM group had higher fasting glucose, HbA1c, total cholesterol, triglycerides, and LDL, but lower FFA (0.69 ± 0.11 vs 2.01 ± 0.55 mmol/L; P<0.001) and HDL than controls (all P<0.001). Mid-pregnancy HIF1A and IGFBP2 were lower in EM, while 1-hour and 2-hour OGTT glucose values were higher (all P<0.001) (**Table 3**).

**Table 3:** Metabolic variables, OGTT values, and circulating biomarkers in mid-pregnancy.

Variables	EM group (n=200)	Control group (n=200)	P-value
FBG (mmol/L)	4.13 ± 0.40	4.62 ± 0.37	<0.001
HbA1c (%)	5.22 ± 0.67	4.66 ± 0.25	<0.001
TC (mmol/L)	6.14 ± 0.46	5.53 ± 0.47	<0.001
TG (mmol/L)	2.54 ± 0.97	1.35 ± 0.20	<0.001
HDL (mmol/L)	2.38 ± 0.35	2.75 ± 0.17	<0.001
LDL (mmol/L)	4.25 ± 0.91	2.49 ± 0.11	<0.001
FFA (mmol/L)	0.69 ± 0.11	2.01 ± 0.55	<0.001
HIF1A (pg/mL)	98.99 ± 9.16	345.25 ± 41.80	<0.001
IGFBP2 (pg/mL)	37.05 ± 16.98	84.51 ± 11.88	<0.001
OGTT 0h (mmol/L)	4.13 ± 0.40	4.62 ± 0.37	<0.001
OGTT 1h (mmol/L)	8.65 ± 1.02	8.13 ± 0.65	<0.001
OGTT 2h (mmol/L)	7.10 ± 1.39	6.24 ± 0.79	<0.001

FBG, fasting blood glucose; HbA1c, glycated hemoglobin; TC, total cholesterol; TG, triglyceride; HDL, high-density lipoprotein; LDL, low-density lipoprotein; FFA, free fatty acid; OGTT, oral glucose tolerance test.

### Incidence of criteria-defined GDM between the two groups

Using IADPSG thresholds applied directly to raw glucose values, GDM occurred in 69/200 (34.5%) EM participants and 27/200 (13.5%) controls (RR 2.56, 95% CI 1.71–3.81; P<0.001). The original workbook labels yielded 65/200 (32.5%) and 25/200 (12.5%), respectively, with a sensitivity-analysis RR of 2.60 (95% CI 1.71–3.95) (**Table 4**).

**Table 4:** Criteria-defined GDM rates between the two groups.

Group	Non-GDM	GDM	P-value
Control group	173 (86.5%)	27 (13.5%)	<0.001
EM group	131 (65.5%)	69 (34.5%)	

Data are shown as n (%). GDM was defined using IADPSG fasting/OGTT thresholds applied to the raw glucose values.

### Long-term temporal coupling of circulating HIF1A and IGFBP2 with maternal glycemic excursions across gestation

To describe the temporal relationship between circulating biomarkers and maternal glycemia, we distinguished the two sampling windows. First-trimester HIF1A and IGFBP2 were measured at 6–7 weeks, before the later 24–28-week diagnostic window, and were inversely correlated with subsequent mid-pregnancy fasting blood glucose (FBG). First-trimester HIF1A was inversely correlated with mid-trimester FBG (r = −0.68, P < 0.01; **Figure 3A**), as was first-trimester IGFBP2 (r = −0.50, P < 0.01; **Figure 3B**). Mid-pregnancy biomarkers and OGTT-based GDM assessment occurred within the same 24–28-week window; because the supplied records do not specify the exact order or interval, the corresponding associations with FBG are contemporaneous and do not establish prediction. Mid-pregnancy HIF1A was inversely correlated with FBG (r = −0.54, P < 0.01; **Figure 3C**), and mid-pregnancy IGFBP2 showed a strong inverse correlation (r = −0.89, P < 0.01; **Figure 3D**).

**Figure 3:**
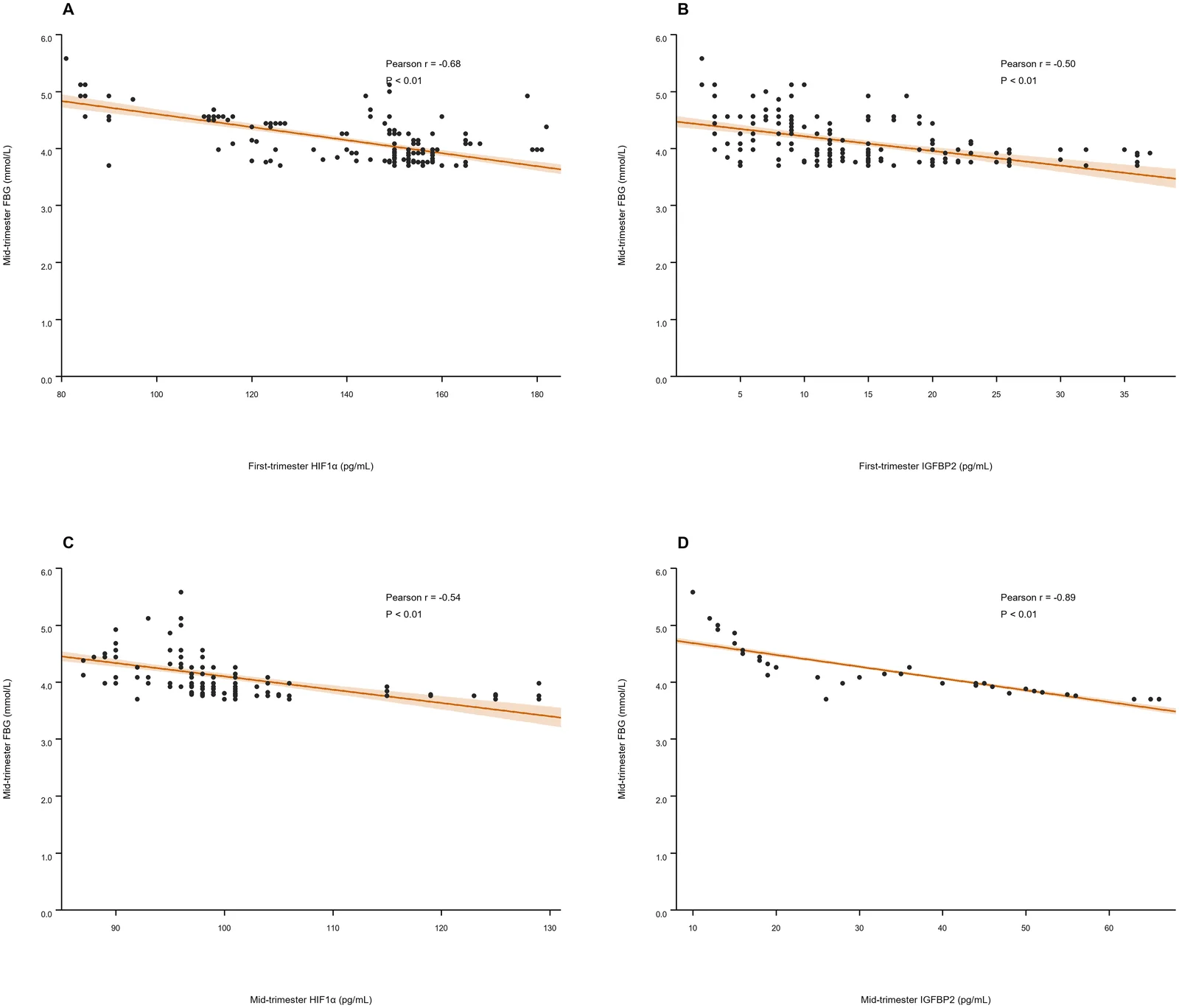
Longitudinal multi-trimester correlation matrices tracking circulating biomarkers against maternal mid-trimester fasting blood glucose in EM patients. **(A)** First-Trimester HIF1A vs. Mid-Trimester FBG: Scatter plot demonstrating a strong, prospective inverse correlation (r = -0.68, P < 0.01) between maternal peripheral blood HIF1A levels quantified during the first trimester and downstream mid-trimester fasting blood glucose (FBG) levels. **(B)** First-Trimester IGFBP2 vs. Mid-Trimester FBG: Scatter plot showing that first-trimester circulating IGFBP2 concentrations were inversely associated with subsequent mid-trimester maternal FBG. **(C)** Mid-Trimester HIF1A vs. Mid-Trimester FBG: Scatter plot validating a synchronized inverse correlation (r = -0.54, P < 0.01) between concurrent mid-trimester circulating HIF1A levels and maternal FBG concentrations. **(D)** Mid-Trimester IGFBP2 vs. Mid-Trimester FBG: Scatter plot revealing an exceptionally tight, powerful inverse coupling (r = -0.89, P < 0.01) between concurrent mid-trimester circulating IGFBP2 availability and maternal FBG concentration.

### Exploratory associations and internal diagnostic performance for criteria-defined GDM

In EM-only age/BMI-adjusted logistic models using criteria-defined GDM, lower early HIF1A (OR 0.922, 95% CI 0.900–0.945) and IGFBP2 (OR 0.920, 95% CI 0.865–0.978) were associated with GDM. Corresponding mid-pregnancy estimates were OR 0.798 (95% CI 0.705–0.903) and OR 0.941 (95% CI 0.909–0.974). Expanded models including parity, early fasting glucose, and early HbA1c gave similar biomarker directions. Individual-marker AUCs were 0.844 (early HIF1A), 0.688 (early IGFBP2), 0.871 (mid-pregnancy HIF1A), and 0.889 (mid-pregnancy IGFBP2). Combined-model AUCs were 0.856 (early) and 0.888 (mid-pregnancy); bootstrap optimism-corrected AUCs were 0.845 and 0.882 (**Figure 4**; **Table 5**). These are exploratory discrimination estimates, and the mid-pregnancy estimates are contemporaneous with the diagnostic window rather than prospective prediction.

**Figure 4:**
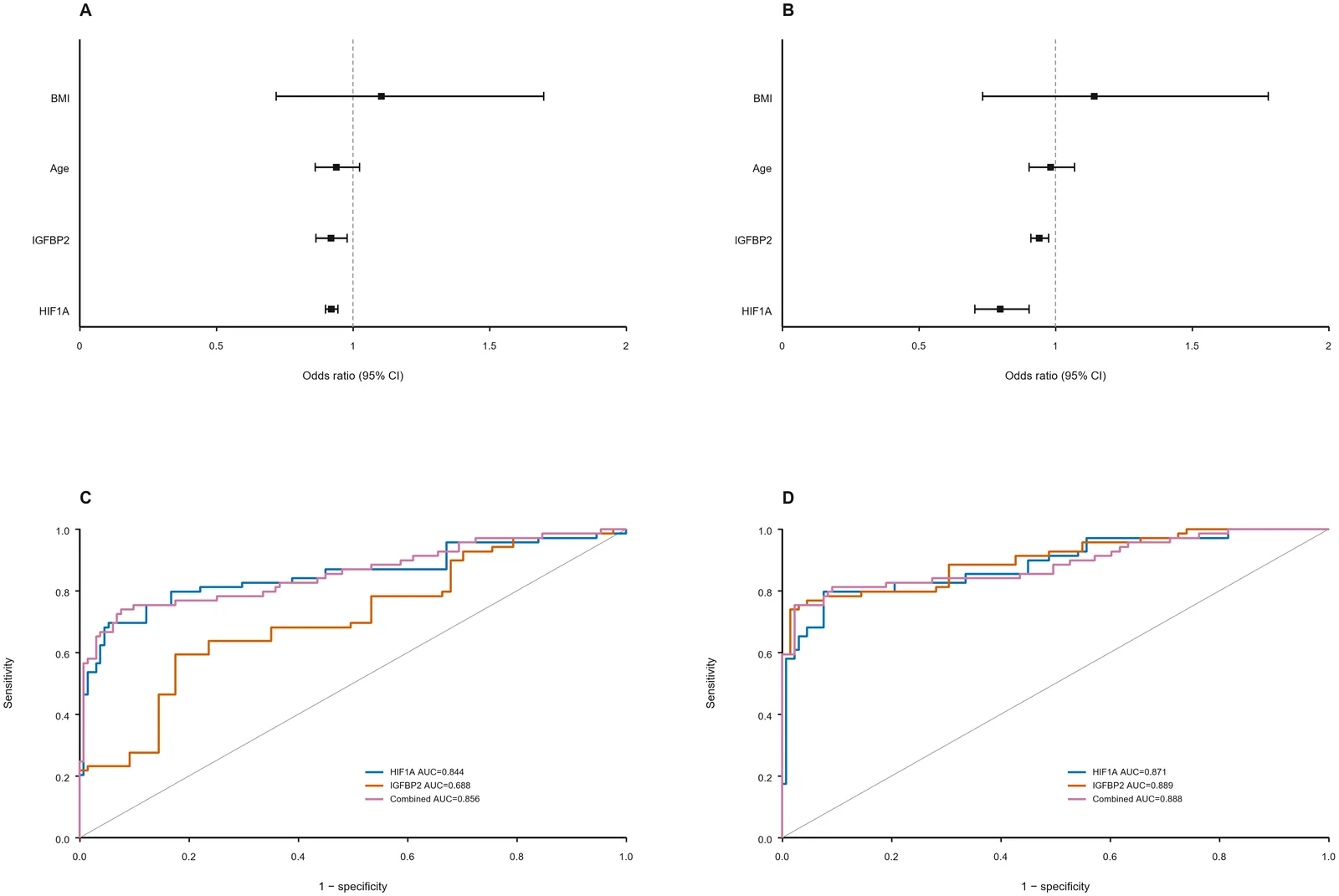
Logistic-regression associations and exploratory ROC performance of circulating HIF1A and IGFBP2 for criteria-defined GDM in the EM cohort. **(A)** Early-pregnancy age/BMI-adjusted odds ratios for HIF1A, IGFBP2, age, and BMI. **(B)** Mid-pregnancy age/BMI-adjusted odds ratios for HIF1A, IGFBP2, age, and BMI. **(C)** Early-pregnancy ROC curves for individual biomarkers and the combined age/BMI-adjusted model. The combined model had AUC 0.856 (95% bootstrap CI 0.791–0.912; optimism-corrected AUC 0.845). **(D)** Mid-pregnancy ROC curves for individual biomarkers and the combined age/BMI-adjusted model. The combined model had AUC 0.888 (95% bootstrap CI 0.829–0.942; optimism-corrected AUC 0.882). Because mid-pregnancy biomarkers and OGTT-based GDM assessment were obtained within the same 24–28-week window, these curves describe contemporaneous exploratory discrimination. ROC CIs were estimated using 2,000 stratified bootstrap resamples. Cutoffs were selected using Youden’s index; sensitivity and specificity are reported with exact binomial CIs.

**Table 5:** Exploratory ROC performance for criteria-defined GDM in the EM cohort.

Marker/model	AUC	95% CI	Cutoff	Sensitivity (95% CI)/Specificity (95% CI)
Early HIF1A	0.844	0.775–0.905	130 pg/mL	69.6% (57.3–80.1)/94.7% (89.3–97.8)
Early IGFBP2	0.688	0.607–0.766	10.5 pg/mL	63.8% (51.3–75.0)/74.0% (65.7–81.3)
Early combined model	0.856	0.791–0.912	p=0.361	73.9% (61.9–83.7)/92.4% (86.4–96.3)
Mid-pregnancy HIF1A	0.871	0.810–0.924	96.5 pg/mL	79.7% (68.3–88.4)/87.8% (80.9–92.9)
Mid-pregnancy IGFBP2	0.889	0.834–0.938	18.5 pg/mL	73.9% (61.9–83.7)/96.9% (92.4–99.2)
Mid-pregnancy combined model	0.888	0.829–0.942	p=0.632	75.4% (63.5–84.9)/97.7% (93.5–99.5)

AUC, area under the receiver operating characteristic curve; CI, confidence interval. CIs are 95% stratified-bootstrap CIs. Combined models include age, BMI, and the two corresponding biomarkers. Sensitivity/specificity use the Youden cutoff. The results have no external validation.

## Discussion

The most pivotal clinical finding derived from our prospective cohort of 400 patients is that a progressive decline in early and mid-trimester peripheral blood levels of both HIF1A and IGFBP2 robustly predicts the subsequent onset of gestational diabetes mellitus (GDM), specifically within the endometriosis (EM) population. Traditionally, gestational metabolic complications are evaluated using conventional maternal risk factors such as advanced maternal age and pre-pregnancy body mass index (BMI) (12–14).

However, after rigorous adjustment for these variables alongside parity, gravidity, and early-trimester metabolic parameters in our expanded binary logistic regression model, both HIF1A and IGFBP2 emerged as highly independent protective factors against GDM. Mathematically, this clinical protective effect dictates that their pathologically driven systemic downregulation serves as a hazardous trigger for compromised maternal glucose tolerance. Crucially, the diagnostic fidelity demonstrated by our receiver operating characteristic (ROC) analysis—yielding an exceptional area under the curve (AUC) for HIF1A and IGFBP2—underscores that these two biomolecules are not merely passive downstream corollaries of metabolic drift. Instead, they act as high-performance upstream prognostic indicators, suggesting a deep-seated pathophysiological linkage between the systemic endocrine-metabolic environment of pregnancy and the cellular dynamics of endometriosis.

A long-standing enigma in reproductive endocrinology is how localized pelvic ectopic lesions managed to cross anatomical barriers and exert systemic, distal subversion over maternal glucose homeostasis during gestation. Historically, conventional bulk-tissue investigations (such as whole-tissue qPCR or Western blot) generated a conflicting narrative, frequently reporting a generalized hyperactivation of HIF1A within endometriosis tissue chunks (15–17).

However, bulk-tissue matrices are heavily confounded by the massive infiltration of reactive stromal components, vascular endothelial cells, and activated immune cell types (e.g., pelvic macrophages), which naturally express elevated levels of HIF1A under inflammatory and hypoxic stimuli, thereby obscuring the true transcriptional state of lesion-specific parenchymal functional cells (18). By harnessing the resolution of single-cell RNA sequencing (**Figure 1**), we successfully bypassed these mixed signals and dissected the distinct cellular compartments within ectopic lesions. Intriguingly, our single-cell data unveiled a profound, cell-type-specific transcriptomic deficiency of both *HIF1A* and *IGFBP2* strictly confined within the functional epithelial and stromal cell lineages of the ectopic niche, while non-parenchymal lineages preserved stress response markers (**Figure 1**). This atypical cell-lineage silencing aligns with the landmark mechanistic framework established by Minet and colleagues, which demonstrated that HIF1A and the insulin-like growth factor (IGF/IGFBP) system operate not as isolated linear paths, but as a reciprocal, tightly coupled positive feedback engine where each node stabilizes and transactivates the other (11). Within our EM cellular cohort, linear regression confirmed an extraordinarily tight co-expression correlation between *HIF1A* and *IGFBP2* (r = 0.42, P = 9e-08; **Figure 2**). Integrating our clinical and transcriptomic datasets, this synchrony implies that the pathological collapse of this coupled feedback engine within pelvic ectopic lesions is mirrored systemically, ultimately manifesting as the dramatic, synchronous depletion of circulating HIF1A and IGFBP2 observed during mid-trimester blood screening.

By stratifying the EM single-cell pool based on endogenous transcript abundance, we unraveled the severe downstream energetic consequences triggered by the disruption of this dual-axis circuit. Global gene set enrichment analysis (GSEA) and target-specific violin plots revealed that IGFBP2_Low lesion cells undergo a catastrophic, multi-pathway paralysis of central carbon processing (**Figure 2**). The transcription of core rate-limiting enzymes governing mitochondrial oxidative phosphorylation—Succinate Dehydrogenase Complex Subunit A (*SDHA*) and Malate Dehydrogenase 2 (*MDH2*)—as well as cytoplasmic glucose processing—Pyruvate Kinase M1/2 (*PKM*) and Lactate Dehydrogenase A (*LDHA*)—was forcefully compressed to baseline zero. From a biochemical perspective, rather than acting as high-capacity oxidative consumers or inducing hyper-glycolytic burn, this cell-type-specific suppression creates an intracellular “metabolic bottleneck” that paralyzes local fuel processing networks, trapping functional ectopic cells in a state of severe intracellular energy starvation.

To reconcile how a localized fuel processing bottleneck manifests as a systemic “metabolic sink” behavior, we emphasize that ectopic lesions operate not as active oxidative consumers, but as “nutrient trappers” and active stress signal generators. Under persistent energy starvation, these pelvic lesions dynamically secrete distinct inflammatory cytokines, anti-angiogenic factors, or exosomal microRNAs into the maternal circulation (19–21). These circulating lesion-derived secretomes orchestrate a distal, compensatory maternal insulin desensitization. This peripheral insulin resistance represents an over-compensatory maternal feedback response designed to forcefully elevate maternal systemic blood glucose levels. By raising the raw extracellular concentration gradient, the maternal system attempts to passively force glucose perfusion back into the starving pelvic lesions to satisfy their metabolic demands and overcome the processing bottleneck. However, when the maternal pancreatic beta-cell reserve fails to keep pace with this persistent, lesion-driven insulin desensitization under the metabolic stress of advancing pregnancy, this systemic deadlock clinically erupts as gestational diabetes mellitus (22, 23).

The synergy between our 400-patient prospective clinical cohort and high-resolution single-cell mechanisms redefines the current clinical paradigm regarding the metabolic cross-talk between endometriosis and maternal homeostasis. Endometriosis has long been clinically characterized primarily as a chronic pelvic pain or infertility syndrome (24). Our data argue that it must be re-classified as a systemic metabolic modifier capable of reshaping maternal gestational health. From a translational perspective, relying solely on traditional risk scoring often fails to identify metabolic vulnerability in non-obese, young EM patients. The exceptional AUC values generated by early-trimester blood screening for HIF1A and IGFBP2 offer a robust, mechanistically backed tool for early risk stratification. Patients identified with low circulating levels of these gatekeepers should be prioritized for early nutritional counseling, continuous glucose monitoring, or prophylactic metabolic interventions. Furthermore, therapeutic strategies aimed at stabilizing the localized HIF1A-IGFBP2 metabolic axis or alleviating the subsequent pelvic tissue energy crisis may hold dual therapeutic promise: suppressing local lesion progression while simultaneously safeguarding maternal systemic metabolic fitness during pregnancy.

Despite the strong clinical and transcriptomic consistency demonstrated in this study, several limitations warrant acknowledgment. First, while our single-cell sequencing and prospective clinical data strongly support the “Metabolic Bottleneck” model, direct spatial protein-level co-localization (e.g., multiplex immunofluorescence or laser-capture microdissection combined with Western blotting) and isolation of exact lesion-derived signaling molecules (such as specific exosomes or cytokines) remain to be fully executed. Future cell-culture and animal models utilizing lesion-conditioned media are required to definitively pinpoint these circulating mediators. Second, while dataset integration via Harmony minimized batch effects, and all dataset donors were hormone-therapy-naïve, subtle variations in lesion microenvironments across original sampling sites could not be completely eliminated. Third, our clinical cohort was derived from a single center, which may restrict the immediate generalizability of the established diagnostic AUC thresholds. Large-scale, multi-center prospective validation trials across diverse ethnic populations are necessary to confirm the diagnostic versatility of mid-trimester circulating HIF1A and IGFBP2 before widespread clinical implementation.

## Conclusion

In this study, women with endometriosis had lower HIF1A and IGFBP2 concentrations during pregnancy and a higher frequency of criteria-defined GDM. Early-pregnancy measurements preceded the later diagnostic window and showed temporal associations with subsequent glycemia, whereas mid-pregnancy measurements were contemporaneous with OGTT-based assessment. This study was the first to revealed the association between abnormal expression of the HIF1A-IGFBP2 axis in patients with endometriosis and the risk of GDM, providing a new perspective on understanding metabolic disorders and pregnancy complications in patients with endometriosis.

## Data Availability

The public single-cell RNA-seq data analyzed in this study are available in NCBI GEO under accession GSE213216 at https://www.ncbi.nlm.nih.gov/geo/query/acc.cgi?acc=GSE213216. The clinical data generated in this study are available from the corresponding author on reasonable request, subject to institutional and ethical restrictions. **Supplementary Table S1** is available with the article.
